# Gastrointestinal Microbiota and Some Children Diseases: A Review

**DOI:** 10.1155/2012/676585

**Published:** 2012-10-30

**Authors:** Thabata Koester Weber, Isabel Polanco

**Affiliations:** Department of Paediatric Gastroenterology and Nutrition, Children's University Hospital “La Paz”, Autonomous University of Madrid, Paseo de la Castellana 261, 28046 Madrid, Spain

## Abstract

The bacterial colonization is defined immediately after birth, through direct contact with maternal microbiota and may be influenced during lactation. There is emerging evidence indicating that quantitative and qualitative changes on gut microbiota contribute to alterations in the mucosal activation of immune system leading to intra- or extra-intestinal diseases. A balance between pathogenic and beneficial microbiota throughout childhood and adolescence is important to gastrointestinal health, including protection against pathogens, inhibition of pathogens, nutrient processing (synthesis of vitamin K), stimulation of angiogenesis, and regulation of host fat storage. Probiotics can promote an intentional modulation of intestinal microbiota favoring the health of the host. This paper is a review about modulation of intestinal microbiota on prevention and adjuvant treatment of pediatric gastrointestinal diseases.

## 1. Composition and Function of Gastrointestinal Microbiota

The microbiota of the human gastrointestinal tract inhabits a complex ecosystem [1]. Normally it is obtained by newborn after birth and suffers influences on the type of delivery and contamination from the environment [2]. At birth, the gastrointestinal tracts are immature and their development continues during the first years of life [3]. During this period, other factors may also influence the colonization of bacteria in the gastrointestinal tract such as the type of milk-feeding (breast-feeding or “formula-feeding”) and possibly genetic characteristics (genotype) [3–5]. 

Facultative anaerobic bacteria, as Enterobacteria, Enterococci, and Streptococci, dominate the first stage of colonization, within a week following birth. After this early stage, these proportions are reversed and the number of strictly anaerobic bacteria, such as Bifidobacteria, *Bacteroides*, and Clostridia, begins to exceed the number of facultative anaerobes bacteria [6]. The commensal bacteria play a substantial role in regulating gut homeostasis [7].

An adequate gut microbial colonization process contributes to the physiological development of the gut and the maturation of the immune system, thereby determining the risk of developing disease later in life [6]. According to Roberfroid et al. [1], several factors can influence the number and diversity of bacteria present in different regions of the gastrointestinal tract. The main factors contributing to variety of bacteria are pH, peristalsis, nutrient availability, oxidation-reduction potential within the tissue, age of host, host health, bacterial adhesion, bacterial cooperation, mucus secretions containing immunoglobulin, bacteria antagonism, and transit time [1]. 

In stomach, the bacterial load is low in healthy individuals. The predominant organisms isolated include Lactobacilli, streptococci, and yeasts [8, 9]. In the duodenum (small intestine) the environment is acidic (pH between 4 and 5), predominating Lactobacilli and Streptococci. In healthy individuals, the number of bacteria in the duodenum is higher than found in the stomach, approximately 10^2^–10^4^ colony forming unit versus 10^2^ CFU. The microbiota changes strongly from the duodenum to the ileum, as the velocity of the intraluminal content decreases and pH increases, increasing bacterial loads to 10^6^–10^8^ CFU. The colon microbiota population reaches 10^10^–10^12^ CFU [8]; an adult may have around 10^14^ CFU, outnumbering the total eukaryotic cells in the human body [10]. 

In this context, there are two factors associated with the greatest physiological colonization in the large intestine: neutral pH and higher transit time. Studies showed that the mean of colonic transit time in pediatric patients without motility disorders is approximately 30 hours [11, 12], higher than in the small intestine, where the transit varies from 2 to 4 hours [1]. 

The main function of the intestinal microbiota is to limit the growth of potential pathogenic microorganisms, preventing the invasion and implementation of these microorganisms on ecosystem. In addition, the microbiota, competes for space and has the capacity to secrete antimicrobial substances (bacteriocins) that inhibit the proliferation of others bacteria. In this process, the microbiota keeps stable, metabolizing subtracts and nondigestible products [1]. Fermentation of nondigestible carbohydrate produces short-chain fatty acids (SCFAs: acetate, propionate, and butyrate), which play an important role in the modulating of different processes in the gastrointestinal tract, including electrolyte, (Ca, Mg, and Fe) and water absorption, cell proliferation and differentiation, hormones secretion, and activation of immune system [8, 13]. Furthermore, the SCFAs are used as energy sources by organism, including colonocytes and a small portion liver and muscle [13]. Metabolic functions are also attributed to microbiota, as vitamin production (K, B12, biotin, folic acid, and pantothenic) and aminoacids synthesis from ammoniac or urea [14].

Currently, it is clear that induction and regulation of the immune system depends on the microbiota. Studies suggest that ability of leukocytes to migrate to the focus of inflammation and to destroy microbial pathogens also could be affected by the SCFAs. According to Vinolo et al. [15] short-chain fatty acids could regulate leukocyte function through cytokines (TNF-*α*, IL-2, IL-6, and IL-10), eicosanoids and chemokines production. 

From a broader context, enterocytes play an important role in the logistics of the immune system, since his position in contact with the intestinal lumen is crucial for the initial recognition of foreign molecules and to generate signals that are transmitted to the immunocompetent cells. The participation of enterocytes in the defense mechanism is not limited only to innate defense. They may act as antigen presenting cells, inducing an acquired immune response mediated by T lymphocytes [16, 17]. Once activated by antigen presenting cells, the expansion of clonal T cell results in helper lymphocytes (Th-cells) of different phenotypes: Th1, Th2, or regulatory T cells. The regulatory T cells play a key role in immune tolerance because they secrete regulatory cytokines, anti-inflammatory tips as IL-10 and TGF-*β*, in response to antigens that are recognized as nonpathogens. This mechanism explains how the immunotolerance behaves when exposed to an innocuous antigenic load, such as foods. On the other hand, defects in the activity of these cells favor the development of diseases due to immune dysregulation [17].

In this paper were prioritized the main intra- and extra-intestinal pediatric diseases which have relation with the gastrointestinal microbiota. Among gastrointestinal diseases were included *Helicobacter pylori* infection, necrotizing enterocolitis, inflammatory bowel diseases, celiac disease, constipation, and diarrhea. Among extraintestinal diseases were included obesity and allergic reactions once they are widely researched and discussed nowadays. 

## 2. Gastrointestinal Microbiota and Diseases

### 2.1. *Helicobacter pylori* Infection

Although the bacterial load in the stomach is low, particular attention has been given to *Helicobacter* species due to their association with various gastric diseases [1]. *Helicobacter pylori* is a gram-negative spiral bacteria that causes gastritis, gastric, and duodenal ulcers, stomach cancer, and mucosa-associated lymphoid tissue lymphoma [18], representing one of the most common bacterial infections in the world [19]. In pediatric patients, this infection is associated with abdominal pain of childhood, gastroesophageal reflux disease, and growth retardation [19]. In adults, recent studies suggest that it increases the risk of coronary heart disease [20]. Overall, over 50% of the world's population is infected by *Helicobacter pylori*, 30%–40% in advanced nations and over 80% in developing countries [21]. Among individuals younger than 20 years old, the prevalence of infection is around 80% in developing countries, higher than in developed countries. Variation in prevalence is associated with sociodemographic factors, such as low socioeconomic status, low-income family, and poor living conditions [18].

### 2.2. Necrotizing Enterocolitis

Necrotizing enterocolitis (NEC) is the most common gastrointestinal medical emergency that occurs in neonates. It represents a significant clinical problem in infants and affects up to 10% of infants who weigh less than 1500 g, especially neonates of extremely low birth weight (<1000 g) with less than 28 weeks of gestation [22]. The prevalence of mortality ranges between 20–30%. The morbidity prevalence is also high, mainly long-term neurodevelopmental impairment in neonates with extremely low birth weight [22, 23]. Despite of advances in neonatal intensive care, NEC continues to be a potentially disastrous illness in preterm neonates, without significant changes in mortality and long-term morbidity “incidence” [23].

The pathogenesis is poorly known. There are several factors including prematurity, hypoxia, formula feeding, especially excess protein substrate in the intestinal lumen, sepsis, intestinal ischemia, and colonization of the intestine by pathogenic bacteria [22, 23]. A recently published study [24] reports higher proportion of Proteobacteria in neonates after diagnosis of NEC, corroborating other findings. Interestingly, in this study, performed on nine neonates patients with NEC and nine in the control group, one week before the diagnosis was found lower proportion of these bacteria in NEC patients compared to the control group [24]. The authors commented that preterm infants with no sufficient colonization of Proteobacteria during the first week of life, may not be able to modulate an adaptative immunological response to subsequent increase of Proteobacteria [24]. Therefore, the tolerance mechanism immaturity, influenced by microbiota quality and quantity can be associated with this pathology. 

### 2.3. Inflammatory Bowel Diseases

In the inflammatory bowel diseases (Crohn's disease and ulcerative colitis), there is an anomalous action in the immune system against elements of microbiota that takes place in intestinal mucosa and causes intestinal damages [25]. Patients with inflammatory bowel disease, in the presence of commensal bacteria, have an increase of antibodies IgG and T lymphocytes presented on mucosa are hyperactive, suggesting the suppression of local tolerance mechanisms [26]. In fact, there are several factors that influence the activation and remission of inflammatory activity, as the derivation of fecal stream, the use of antibiotics to treat Crohn's disease and broad-spectrum antibiotics in light of the colon-to-ulcerative colitis [27, 28]. Considering that Crohn's disease in humans occurs in the terminal ileum and colon, where the bacterial concentration is higher [26], it is assumed that the intolerance generated by the microbiota, added to genetic predisposition, favors the development of this inflammation [25].

The lamina propria is the tissue site where immune cells initially recognize bacterial antigen, prior to migrating to the distal lymphoid tissue to mount the inflammatory response [29]. Epithelial cells present receptors named Toll-like receptors and NOD2 receptors that are important to start immune response. Once activated, these receptors can generate an intracellular response, producing proinflammatory cytokines [30]. This mechanism favors mucosal dendrite cells maturation that after contact with antigen follows to local lymphoid structures, such as Peyer's patches, and draining mesenteric lymph nodes to initiate or maintain T- and B-cell immune responses. Dendrite cells initiate immune response, control intestinal inflammation, and maintain the tolerance. In this context, mucosal dendrite cells are assumed to play key roles regulating immune responses in the antigenic gastrointestinal environment, maintaining intestinal homeostasis and allowing the pacific coexistence with the endogenous microflora [29]. In normal subjects, the commensal flora is unable to cross the epithelial barrier; however, when any of these bacteria pass through intestinal barrier, are quickly phagocytosed by macrophages of the mucosa, avoiding the activation of intestinal immune response; but, on the other hand, when pathogens microorganims cross the barrier, this response is activated [31]. 

Ulcerative colitis and Crohn's diseases are generally developed in areas with the highest concentration of enteric microbiota, suggesting that commensal bacteria associated to a genetic susceptibility can contribute to the pathogenesis of these diseases [32]. Furthermore, several studies cited by Fava and Danese [33] suggest that microbiota found in patients with inflammatory bowel disease is different from healthy individuals, suggesting that dysbiosis may lead to intolerance. These patients present a poorly diversified microbiota, with prevalence of Clostridia, *Bactoroides*, and Bifidobacteria (commensal microbiota) and a concomitant increase in detrimental bacteria, such as *Escherichia coli*, uncommon in individuals without inflammatory bowel disorders [33]. The proportion of detrimental bacteria could represent 30–40% of dominant bacteria, although significant, the relation of cause and effect is not well established [34]. It is interesting to mention that the low diversity of species is related to instability in the ecosystem, which implies a greater predisposition to change the composition by environmental influences. Thus, ecosystem instability may favor the risk of inflammation. 

### 2.4. Celiac Disease

Celiac disease is an autoimmune disease of the small intestine, characterized by life-long intolerance to the gliadin and related prolamins from wheat and other cereals that occurs in genetically predisposed individuals to gluten intolerance [35]. Through intolerance mechanisms to gluten are activated both immune responses: (1) adaptive immune response, dominated by Th1 pro-inflammatory cytokines and (2) innate immune response, mediated by interleukin-15, that result in epithelial alterations, with changes in permeability and integrity of intestinal mucosa, favoring active phase of the disease [36]. In response to immune activation is developed atrophy of intestinal villous with different degrees, until the complete villous atrophy, associated with an increase in crypt length and cells number [35]. This abnormality in the mucosal structure may lead to nutrient malabsorption, failure to thrive, osteopenia, osteoporosis, extraintestinal autoimmune disorders as herpetiform dermatitis, infertility and neoplastic processes, which can be prevented with the exclusion of gluten from the diet. Thus, a strict gluten-free diet is the only therapy for remission of the clinical symptoms and health complications in these patients [35, 37].

The role of microbiota in celiac disease is not completely understood in comparison with healthy individuals [38]. Genetic factors were associated with the colonization of *Bacteroides* species [39]. Studies showed that a strict gluten-free diet, in celiac patients, promotes reduction in beneficial bacterial counts, especially *Bifidobacterium* and *Lactobacillus* compared to gram-negative bacteria (*Bacteroides* and *E*. *coli*) [36]. This microbiota alteration due to gluten exclusion may result from the exclusion of important carbohydrates sources, the main energy source for commensal microbiota [39]. The dietetic treatment for these patients does not favor completely the intestinal homeostasis; however, the immune suppressive response by microbiota may be beneficial for celiac disease patients [40]. 

It is estimated that over 1 in every 100 newborn babies will develop the disease throughout his life. In the past, it was thought that celiac disease rarely appears in childhood, nowadays, is known as a relatively common disease that can be diagnosed at any age. This is because the prevalence of typical forms of the disease are higher in children than in adults, respectively, 67.0% versus 14.3% of the cases [41], but up to 20% of the patients are diagnosed after 60 years old. Currently are observed great advances in the celiac disease diagnosis that will benefit patients and their families, as also asymptomatic individuals [37]. Parallel to this, advances in knowledge of the microbiota may benefit the disease prevention.

### 2.5. Constipation

Chronic constipation is the main complaint on pediatrics consults reaching 25% of gastroenterology services [42, 43]. It is a common condition in pediatric patients with multifactorial etiology, being 90–95% due to functional causes [44]. Rome III criteria define chronic constipation as the presence of at least two of the following symptoms for two or more months: (1) two or fewer defecations per week; (2) at least one episode of fecal incontinence per week; (3) history of retentive posturing or excessive volitional stool retention; (4) history of painful or hard bowel movements; (5) presence of a large fecal mass in the rectum; (6) history of wide diameter stools that may obstruct the toilet [43]. 

Zoppi et al. [45] with the aim of investigating the composition of the intestinal ecosystem in chronic functional constipation found dysbiosis in constipated children (mean of age 8.6 ± 2.9 years). In this study was observed a higher number of Clostridia and Bifidobacteria in feces than in healthy children. Evaluating infants, Aguirre et al. [46] found that artificial feeding increases by 4.5 times the risk of constipation in relation to predominant breastfeeding. In breast-fed infants, *Bifidobacterium spp.* predominate, representing up to 90% of the total fecal microbiota, whereas in formula-fed infants the microbiota is more heterogeneous [47, 48]. Breast-milk has been shown to be a continuous source of commensal bacteria to the infant gut, maybe because the prebiotics composition that stimulates growth and maintenance of *Lactobacillus* and *Bifidobacterium* [47, 48]. The addition of prebiotics to artificial infant formula increases the bifidobacteria counting [49], the stool frequency and decreases stool consistency, similar characteristics than found in breast-fed infants [50, 51]. 

The presence of commensal bacteria in the colon promotes the reduction of pH which stimulates further growth, due to its high percentage of water, that provides more humid stool [52, 53] and lower consistency, which facilitate their excretion [54].

### 2.6. Diarrhea

Diarrhea is a symptom defined as the loose of watery unformed stools for more than three times in one day or defecation volume greater than 200 mL/day. The acute diarrhea is the main symptom of acute gastroenteritis. According to ESPGHAN (European Society for Paediatric Gastroenterology, Hepatology, and Nutrition), acute gastroenteritis is defined as the decrease in the feces consistency (soft or liquid) and/or an increase of stool frequency (3 or more times in 24 hours) with or without fever or vomiting, usually lasting less than 7 days and never exceeding 14 days [55]. In some cases, it is accompanied by abdominal pain and dehydration [56].

In Europe, the incidence of diarrhea varies between 0.5 and 1.9 episodes per year among children under 3 years old [55]. A recent systematic review, including 139 low- and middle-income countries, estimated that the incidence of diarrhea declined from 3.4 episodes/child per year in 1990 to 2.9 episodes/child per year in 2010, being highest among infants 6–11 month of age (4.5 episodes/child per year in 2010) [57]. Although there was a reduction on the incidence of episodes per child over the years, the number of episodes is still high, nearly 1.7 billion reported in 2010 [57].

The most common etiologic agents are Rotaviruses and, among bacteria, *Campylobacter* is the most common, followed by *Salmonella*. Parasites such as *Giardia lamblia* and *Cryptosporidium* are an infrequent cause of diarrhea in healthy children. The main etiologic agents can change with the child age: in children under 1 year, Rotavirus, Norovirus, Adenovirus, and *Salmonella* prevail. From 1 to 4 years old the same agents prevails, adding *Campylobacter* and *Yersinia*. In children older than 5 years, *Campylobacter*, *Salmonella*, and Rotavirus [55]. Diarrhea associated with antibiotics administration is also a frequent, reaching an incidence up to 30% [58]. 

The main pathogenic mechanism of gastroenteritis, regardless if its cause, is changes in the absorption and secretion of water and electrolytes through the intestinal mucosa, it increases, especially in infants, the risk of developing acute dehydration, considered the main complication of gastroenteritis [59]. In this sense, a modulation of the intestinal microbiota can help to control symptoms and complications of diarrhea as part of an adjuvant treatment. 

## 3. Intestinal Microbiota and Extraintestinal Diseases

The intestine is the first barrier for nutrients and luminal components, constituting one of the first lines of defense against infectious agents and allergens [26]. Bacterial translocation is caused by increases intestinal permeability as a result of a disrupter's intestinal barrier [60]. As previously mentioned, alterations of intestinal microbiota can be related to the development of extraintestinal diseases as obesity and allergy reactions.

### 3.1. Obesity

Nowadays, obesity is becoming a serious public health problem. The prevalence of childhood obesity can reach 20% in European Union countries, and is increasing in developing countries [61]. The impaired balance between energy intake and energy expenditure is the main risk factor to excessive weight gain in children and adolescents. 

Recent studies suggest differences in the quantity and quality of microbiota in obese and lean children as mentioned by Angelakis et al. [62]. In a prospective followup, Kalliomäki et al. [63] observed that children with normal weight at 7 years presented higher prevalence of *Bifidobacterium* species during the first year of life than those with overweight. The presence of bacteria in the newborn is influenced by maternal bacteria and subsequently by exposure to breast milk or type of feeding adopted [62]. 

Before pregnancy, overweight women have a higher number of Bacteroides than women with normal weight. In addition, an increase in *Bacteroides* number during pregnancy is associated with excessive weight gain [64]. In relation to the reduction or increase in the number of *Bacteroides* in obese and not obese adults, studies are controversial, but many of them, conclude that *Bacteroides* increases in obese, including individuals with diabetes [62]. In an experimental animal model, an increase of *Bacteroides* in the gut microbiota was associated to predisposition toward energy storage and obesity [63]. In adults, a recent meta-analysis, did not find any difference in the *Bacteroides* concentration between obese and normal-weight individuals [62]. 

Among children, a high intestinal *Bacteroides fragilis* and low *Staphylococcus* concentration, when infants, were associated with higher BMI in preschool children [65]. On the other hand, children with excessive weight at 7 years old did not present significant increase in the number of *Bacteroides* when compared to normal-weight children at the same age [63]. In adolescents and adults, a significant increase in the ratio of *Bacteroides* was correlated to weight loss, after the weight lost program [62]. 

Until this moment, the main association of intestinal microbiota with obesity is related to Bifidobacteria presence. A meta-analysis performed with 159 obese versus 189 control subjects, from six published studies, demonstrated a significant Bifidobacteria depletion in obese group [62]. Other meta-analysis showed that microbiota of obese individuals presents lesser Firmicutes and *Methanobrevibacter spp*. concentration than normal weight individuals [62]. Thus, the studies suggest a causal relation between microbiota and obesity. 

### 3.2. Allergic Reactions

In occidental countries, the prevalence of alimentary allergy can affect 6–8% of children lower than 10 years old and 1–4% of adult population. The alimentary allergic manifestation can occur anywhere in the body, including gastrointestinal tract, skin, and respiratory system [66]. Several studies show an increased intestinal permeability in patients with allergies, which facilitates the passage of proteic antigens coming from diet [67]. In addition, epidemiological studies demonstrated that allergic children have a different microbiota from healthy children with higher levels of Clostridia and lower levels of Bifidobacteria [68]. On the other hand, in nonallergic children, in the composition of the microbiota are found more commonly Bifidobacteria and Lactobacilli, suggesting that modulation of the microbiota can help to promote prevention of allergic manifestation [68, 69]. 

Most of publishes about modulation of intestinal microbiota in allergic patients are focused on dermatological pathology, especially in atopic dermatitis. It is a chronic disorder in the skin that can affect from 10% to 25% of occidental children [70]. Genetics is not the only risk factor, the “hygiene hypothesis” proposes that a quick increase in atopic manifestations may be due to less exposure to infections in the first stage of life. Following this theory, a relative lack of microbial stimulation of the infantile gut immune system and the exaggerated hygiene from the typical western lifestyle during early childhood, influencing the immune maturation [68]. 

Development of the child's immune system tends to be directed towards a T helper type 2 (Th2) phenotype in infants, whereas postnatal maturation is associated with gradual inhibition of Th2 and increasing Th1 affinity. According to Prescott et al. [71], nonatopic children during the first year of life present rapid suppression of Th2 responses, while the response in atopic children is stimulated. The predominant Th2 response is associated with increased production of IgE antibodies against environmental antigens and eosinophilia, forming the basis of allergic processes.

Considering microbiota and atopic reactions, other studies also showed that early colonization with potentially more pathogenic bacteria such as *Clostridium difficile* and *Staphylococcus aureus* is more likely to occur in children who go on to develop allergy [68].

## 4. Modulation of Intestinal Microbiota

Scientific advances leads every day to more knowledge on the microbiota and its association with pediatric diseases. In the face of published studies is seeking alternatives for modulation of intestinal microbiota, in order to prevent and treat a wide range of diseases. At present, the probiotic use is being considered a therapeutic strategy due to its ability to inhibit pathogenic bacteria overgrowth and the development of inflammatory intestinal infections [72]. Probiotics are defined as “live microorganisms that when administered in adequate amounts confer a health benefit to the host” [8]. For a microorganism strain to be considered beneficial to organism or probiotic needs to fulfill the following criteria: (1) remains viable at the intestine, resisting gastric acid digestion and bile salts; (2) adheres to the gastrointestinal epithelium; (3) be metabolically active; (4) decreases colon pH; (5) shows antibacterial activity against pathogenic bacteria [72]. *Lactobacillus plantarum*, *L. rhamnosus*, *L. reuteri*, *L. casei*, *L. acidophilus*, *Bifidobacterium longum*, *B. brevis*, and *B. bifidum* are the main strain with proven probiotic effects. The benefits of a probiotic action vary according to interval time that the bacteria remain in the intestine and their quantity [73]. 

Although the amount and time of administration are important for the beneficial effects, its recommendation requires attention. The most important concern about probiotic use is the risk of sepsis [72]. Probiotics have specific actions to specific patients. So, it is important to emphasize that probiotics are highly heterogeneous with differences in composition and biological activity [73].

In the following are addressed the indications of probiotic to intestinal microbiota modulation for each disease presented in this paper.

### 4.1. *Helicobacter pylori*

Studies *in vitro* showed that *L. acidophilus* LB associated with antibiotics therapies exerts an agonist effect against the bacteria, affecting negatively growth of bacteria, inhibiting adhesion receptor glycolipids, and decreasing the activity of the urease, essential for their survival in acid environment [74]. In humans, the probiotic strain can improve infection condition, but not enough to *H. pylori* eradication, as demonstrated in a recent published review about *Helicobacter pylori* infection and childhood [19]. The authors state that the environment where children live is important to prevent this infection. 

The use of supplementation with fermented milk, containing probiotic *L. casei* DN-114 001 (Actimel) for 14 days, benefited the *H. pylori* eradication in children treated with omeprazole, amoxicillin, and clarithromycin during 7 days [75]. This result suggests that the probiotic could be used as adjuvant treatment for eradication of HP in children.

### 4.2. Necrotizing Enterocolitis

The evaluation of the probiotics use to reduce the risk of NEC are based in two recent meta-analyses. In the first meta-analysis mentioned was selected 11 trials using a fixed-effect model, with preterm very low-body-weight neonates (<34 weeks of gestation and birth weight <1500 g). An enteral administration of any probiotic commenced within the first 10 days of life and continued for at least 7 days, allowed 30% reduction in the incidence of NEC. In addition was demonstrated in supplemented preterm neonate a lower risk of death [23]. In the second meta-analysis, enteral probiotics supplementation also reduced the incidence of severe NEC (RR 0.32; 95% IC 0.17–0.6) and mortality (RR 0.43; 95% IC 0.25–0.75), in preterm neonates (<37 weeks of gestation and/or <2,500 g birth weight) [76]. In both studies, the use of probiotics does not decrease the risk of sepsis. 

The studies found significant variation in probiotics strains and protocols treatment, data suggest that additional placebo-controlled trials are necessary. According to Deshpande et al. [23] if offered as a routine therapy for preterm neonates a strict product selection should be made, including a close monitoring of the target population before the consumption. Although the results are positive, the ESPGHAN Committee on Nutrition still does not recommend the routine use of probiotics in order to prevent NEC [77]. 

### 4.3. Inflammatory Bowel Diseases

Probiotics can adjust the metabolic activity of the intestinal flora and their components by preventing bacterial overgrowth, maintaining the integrity of the intestinal mucosal barrier, improving their function, permeability, stability, and induction of T-cell apoptosis. Through this action mechanism, the probiotics can regulate the immune response and reduce the secretion of pro-inflammatory factors [4, 8]. 

In ulcerative colitis treatment, the use of *Escherichia coli* Nissle, 1917, nonpathogenic, was evaluated 116 patients aged 18–80 years old in a randomized clinical trial controlled with mesalazine. The authors found a 74.6% reduction of remission in patients that consumed mesalazine versus 68.4% in patient with probiotic consumption (OR 1.35; 95% IC 0.6–3.04). In this study, the time to remission was similar, respectively, 44 and 42 days [78]. On the other hand, a meta-analysis published in 2010, selecting 13 randomized controlled studies, suggests that the probiotic use did not provide additional benefit in an induction remission, although in the remission maintenance of symptoms, the probiotic 1.36 (95% CI 1.07–1.73) was more effective than placebo 0.69 (95% CI 2.47–1.01) [79]. Considering controlled studies, *Escherichi coli,* Nissle, 1917 (2.5 × 10^10^) [78, 80], Bifidobacteria (bifidobacteria-fermented milk 100 mL/day) [81], and Lactobacilli GG (18 × 10^9^) [82] were probiotics that resemble mesalazine effect in the maintenance treatment of remission. 

 In the treatment of active and inactive Crohn's diseases, many studies with several probiotics strain (*E. coli* Nissle, LGG, *L. johnsonii* LA1, *S. boulardii* y VSL *number* 3) were performed, but the sample size was small. Furthermore, these studies, as well as in experimental animal studies [83], the results showed no significant difference in the reduction of inflammation [84]. In the open label pilot evaluation using *Lactobacillus* GG (10^10^ CFU) twice a day for 6 months, 3 of 4 children were reported to have improved Pediatric Crohn's Disease Index (PCDAI) scores. But more randomized controlled trials are required to assess the efficacy of *Lactobacillus* GG in children Crohn's disease as well as evaluate other probiotics strains [85].

### 4.4. Celiac Disease

 Currently, there is no probiotic indication as adjuvant treatment for celiac diseases. However, the advancement of microbiota knowledge will allow future investigations, especially about microbiology and genetic predisposition. 

The major genetic risk factor on celiac disease is represented by human leukocyte antigen (HLA)-DQ genes [35]. The relation between milk-feeding type and HLA-genotype on intestinal microbiota composition of infants with a family history of celiac disease was recently studied. Palma et al. [6] published the first study that found this association. According to the authors, infants with high genetic risk of developing celiac disease presented a higher number of *B. fragilis* and *Staphylococcus spp.*, and lower number of *Bifidobacterium spp*. and *B. longum*. Breast-feeding reduced the genotype-related differences in microbiota composition, which could partly explain the protective role attributed to breast milk in this disorder. Epidemiological studies suggest that breast-feeding confers a protective effect against the risk of celiac disease development, mainly when gluten is introduced in the diet while the infants are still breast-fed [86]. Therefore, the protective effect of breast-feeding is also related to its ability to modulate the intestinal microbiota in infants, favoring the increase and maintenance of Bifidobacteria. 

### 4.5. Constipation

A recent systematic review concerning nonpharmacological treatments for childhood constipation considers poor the evidence about probiotics effects [87]. A review on adults and children indicates that the use of probiotics for the constipation treatment should be considered investigational [88]. In adults, studies demonstrate beneficial probiotic effect compared to placebo, as can be seen in a crossover study with *Lactobacillus casei* Shirota (0.5–5 × 10^9^ UCF) [89] and in a randomized control trial with *E. coli* Nissle, 1917 (6.5 × 10^9^ UCF) [90]. In pediatric patients, the positive probiotic effect in functional constipation was observed with different probiotic strains. The yogurt supplemented with 10^9^ CFU/mL *Bifidobacterium longum* compared with placebo presented significant differences for defecation frequency, defecation pain, and abdominal pain in a crossover, double-blind controlled trial that included 59 children and adolescents aged 5–15 years old [91]. The treatment with *Lactobacillus casei* rhamnosus Lcr35 (8 × 10^8^ CFU/day) presented no difference in efficacy compared with magnesium oxide (50 mg/kg/day), being the occurrence of abdominal pain less prevalent in the Lcr35 group [92]. In a pilot study to evaluate efficacy of a probiotic mixture (Ecologic Relief: Bifidobacteria *B. bifidum, B. infantis, B. longum, Lactobacilli casei, L. plantarum* and *L. rhamnosus*) in pediatric patients aged 4–16 years during 4 weeks, it was observed a significant effect of this mixture (4 × 10^9^ CFU), increasing stool frequency and decreasing the number of fecal incontinence and abdominal pain [93]. On the other hand, the *Lactobacillus* GG as an adjunct to lactulose for the treatment of functional constipation in children presented no positive effect [94], but in this case, the laxative effect of lactulose should be considered. In general, the probiotic use seems to benefit the functional constipation in pediatric patients, but a larger number of cases and a longer followup are required to establish a conclusion. 

 In normal healthy infants (under 6 months of life), a randomized controlled trial demonstrated a significant increase in stool frequency and fecal consistency improvement using a LGG-supplemented formula [95]. Contradictory, the administration of *B. lactis*, *B. longum* BL 999, and rhamnosus LPR was not associated with change in stool frequency and stool consistency, resulting unclear the probiotic effect in infants according to ESPGHAN commentary published in 2012 [77].

### 4.6. Diarrhea

The probiotics use can benefit the reduction of diarrheal symptoms through multiple action mechanisms. Firstly, it can preserve the function of the intestinal epithelium, avoiding the increased intestinal permeability [96] and the invasion of pathogenic microorganisms [97]. Secondly, through colon acidification, bactericides production, competition for nutrients, and IgA production, it prevents the growth of pathogenic strains [98]. Finally, the production of short chain fatty acids in the colon stimulates the absorption of sodium by the colonocytes [8]. *Lactobacillus* strains and *Saccharomyces boulardii*, a type of yeast, are the most studied microorganisms. 

Several meta-analyses [58, 99–102] confirmed the beneficial effects of *Lactobacillus* GG and *Saccharomyces boulardii* in adjuvant treatment of diarrhea. Although the randomized clinical trials realized with *Saccharomyces boulardii* presented a high methodological variability, they show positive results. In a large meta-analysis, it was verified that this yeast reduced the risk of diarrhea at 3 days (RR 0.66; 95% IC 0.55–0.77), and on average reduction of 30.48 h (95% IC 18.51–42.46 h), proving its effective action on rehydration treatment [103]. 

 In antibiotic therapy, the auxiliary supplementation of *Saccharomyces boulardii* decreased the risk of diarrhea from 17.2% to 6.7% (RR 0.43; 95% IC 0.23–0.78) in children and adolescents between 6 month and 14 years old [58]. In another revision including pediatric patients (1015 subjects treat and 971 control), the authors observed a significant reduction in the incidence of antibiotic-associated diarrhea with probiotic administration (RR 0.49; 95% IC 0.32–0.74) [104]. In a more recent study, including 3432 children and adolescents between 0–18 years of age, positive results were observed to probiotics, but in a subgroup analysis was indicated that higher doses (≥5 billion CFU/day) are more effective than lower probiotic doses (<5 billion CFU/day) (*P* = 0.010) [105]. According to Johnston et al. [105], the incidence of antibiotic-associated diarrhea for high-dose studies in the probiotic and placebo groups was, respectively, 8% and 22% (1474 participants; RR 0.40; 95% CI 0.29–0.55). While for low-dose studies was found an incidence of 8% in the probiotic group and 11% in the control group (1382 participants; RR 0.80; 95% CI 0.53–1.21) [105]. 

In children with acute diarrhea, the *Lactobacillus* GG supplementation is linked to a significant decrease of diarrhea duration, especially caused by rotavirus and diarrhea with lower seven-day risk. Although no percussion in feces volume was observed, its administration was associated with a moderate benefit [100].

In hospitalized children, the consumption of *Lactobacillus* GG compared with placebo has the potential effect to reduce the overall incidence of healthcare-associated diarrhoea (RR 0.37; 95% CI 0.23–0.59), including symptomatic rotavirus gastroenteritis (RR 0.49; 95% CI 0.28–0.86) [102]. The *Saccharomyces boulardii* reduced the duration of diarrhea in hospitalized patients with acute infectious diarrhea [106]. Dinleyici et al. [106] found a reduction of approximately 24 h in diarrhea duration and a 20 h reduction in hospitalization, a significant impact on clinical practice. 

There is a strong evidence of the clinical benefit of *Lactobacillus* and *Saccharomyces boulardii* [107] to diarrhea treatment. About the efficacy and safety of other probiotics agents on pediatric diarrhea treatment, it is premature to draw conclusions. Further studies to evaluate the dose effects and its safety are demanded [105].

### 4.7. Obesity

For obesity, no auxiliary treatment with probiotics is recommended. Previously several short-term randomized controlled trials showed the benefit of probiotics to insulin sensitivity, inflammatory markers and glucose tolerance [108]. Considering that Bifidobacteria depletion during lactation is an associated factor to obesity development in school age [63], and the first years of life have a crucial impact on the individual's gut microbiota composition, studies have been developed to evaluate the probiotic supplement during prenatal and postnatal periods [108].

The impact of perinatal probiotic intervention on the development of overweight during a 10-year followup was evaluated by Luoto et al. [109]. In this study, the consumption of 10^10^ CFU of *Lactobacillus* GG 4 weeks before expected delivery extended for 6 months postnatally appeared to moderate the excessive weight gain especially among children until 24–48 months. *Lactobacillus* GG and *Bifidobacterium lactis* administrated during pregnancy reduced the risk of gestational diabetes mellitus and decreased the risk of larger birth size in affected cases [110]. Currently, the findings in pediatric subjects are limited. Although studies demonstrated an association between obesity and type 2 diabetes with specific changes in intestinal microbiota composition, mainly in animal model [111], further studies to human extrapolation are necessary. 

### 4.8. Allergic Reaction

 There are many studies that investigated the role of probiotics in atopic dermatitis in children younger than 2 years old. The offering of *Lactobacillus* GG compared with placebo reduced by half the risk of atopic eczema in children genetically susceptible in the first year of life (RR 0.51; 95% CI 0.32–0.84) [112]. In this study, both administration forms were effective: by infant, during 6 months postnatal or by his mothers, during pregnancy. Other studies demonstrated a significant reduction of severity scoring of atopic dermatitis index using *Lactobacillus fermentum* VRI-033 PCC 1 × 10^9^ CFU twice a day during 8 weeks [113] and *Lactobacillus* GG in infants suspected cow's milk allergy IgE-sensitized [114]. In children older than 2 years was also found significant difference on decreasing of severity scoring of atopic dermatitis index with *Lactobacillus* GG [115] and *Lactobacillus reuteri* [116] supplementation when compared to placebo. 

A meta-analysis published in 2012 based on 14 studies provided evidence to support a moderate role of probiotics use in atopic dermatitis. Probiotic supplementation decreased the incidence of atopic dermatitis (RR 0.79; 95% CI 0.71–0.88) and IgE-mediated atopic dermatitis in infants (RR 0.80; 95% CI 0.66–0.96), when used during pregnancy or early life [117]. Furthermore, it is important to comment that breast-feeding may protect against infection and atopy in infants, delivering IgA and other protective molecules TGF and IL-10 (with anti-inflammatory properties) and favoring Lactobacilli and Bifidobacteria increase [66].

Considering respiratory symptoms, the *Bifidobacterium lactis* is the most common probiotic studied in children. Used in infant born at term which mother diagnosed with HIV, the *B. lactis* CNCM I-3446 demonstrated no significant effect in the rate of bronchopneumonia (RR 0.75; 95% CI: 0.2–2.6) [118]. When tested in infants from 4 to 10 month of age, *B. lactis*, as *L. reuteri* also did not present effect on the rate and duration of respiratory illness. In this study was observed that infants fed with formula control had more diarrhea episodes and episodes of longer duration [119]. 

 The infants formula supplemented with *Lactobacillus salivarius* CECT5713 (2 × 10^6^ CFU) provided a significant reduction in the number of episodes of respiratory infections compared with control formula, respectively, 53 versus 36 after 6 months of consumption [120]. The use of *L. johnsonii* La1-supplemented formula did not show any positive effect on the number of respiratory infections compared to the control formula [121]. According to ESPGHAN Committee on Nutrition, available data are insufficient to draw a reliable conclusion about using probiotic supplement formula on prevention of respiratory infections in infants [77].

## 5. Conclusion

The key role in human health could depend on homeostasis balance among microbial species populating the gut. Therefore, the intestinal microbiota is considered not only a potential biomarker, but also an important factor in the adjuvant therapeutic a therapeutic target in intra- and extra-intestinal diseases. Although all people are genetically programmed, these changes in gastrointestinal microbiota can be modulated. Nevertheless, the type of bacteria, dosing regimen, delivery method, and the host should be considered when indicated a probiotic supplementation.
